# Excitatory Synaptic Input to Hilar Mossy Cells under Basal and Hyperexcitable Conditions

**DOI:** 10.1523/ENEURO.0364-17.2017

**Published:** 2017-12-04

**Authors:** Tristan P. Hedrick, William P. Nobis, Kendall M. Foote, Toshiyuki Ishii, Dane M. Chetkovich, Geoffrey T. Swanson

**Affiliations:** 1 Department of Pharmacology, Northwestern University Feinberg School of Medicine, Chicago, IL 60611; 2 Department of Neurology, Northwestern University Feinberg School of Medicine, Chicago, IL 60611; 3 Department of Physiology, Nippon Medical School, Tokyo 113-8602, Japan; 4 Department of Neurology, Vanderbilt University Medical Center, Nashville, TN 37232

**Keywords:** aging, CA3, hilus, mossy cell, seizure

## Abstract

Hilar mossy cells (HMCs) in the hippocampus receive glutamatergic input from dentate granule cells (DGCs) via mossy fibers (MFs) and back-projections from CA3 pyramidal neuron collateral axons. Many fundamental features of these excitatory synapses have not been characterized in detail despite their potential relevance to hippocampal cognitive processing and epilepsy-induced adaptations in circuit excitability. In this study, we compared pre- and postsynaptic parameters between MF and CA3 inputs to HMCs in young and adult mice of either sex and determined the relative contributions of the respective excitatory inputs during *in vitro* and *in vivo* models of hippocampal hyperexcitability. The two types of excitatory synapses both exhibited a modest degree of short-term plasticity, with MF inputs to HMCs exhibiting lower paired-pulse (PP) and frequency facilitation than was described previously for MF–CA3 pyramidal cell synapses. MF–HMC synapses exhibited unitary excitatory synaptic currents (EPSCs) of larger amplitude, contained postsynaptic kainate receptors, and had a lower NMDA/AMPA receptor ratio compared to CA3–HMC synapses. Pharmacological induction of hippocampal hyperexcitability *in vitro* transformed the abundant but relatively weak CA3–HMC connections to very large amplitude spontaneous bursts of compound EPSCs (cEPSCs) in young mice (∼P20) and, to a lesser degree, in adult mice (∼P70). CA3–HMC cEPSCs were also observed in slices prepared from mice with spontaneous seizures several weeks after intrahippocampal kainate injection. Strong excitation of HMCs during synchronous CA3 activity represents an avenue of significant excitatory network generation back to DGCs and might be important in generating epileptic networks.

## Visual Abstract

**Figure:**
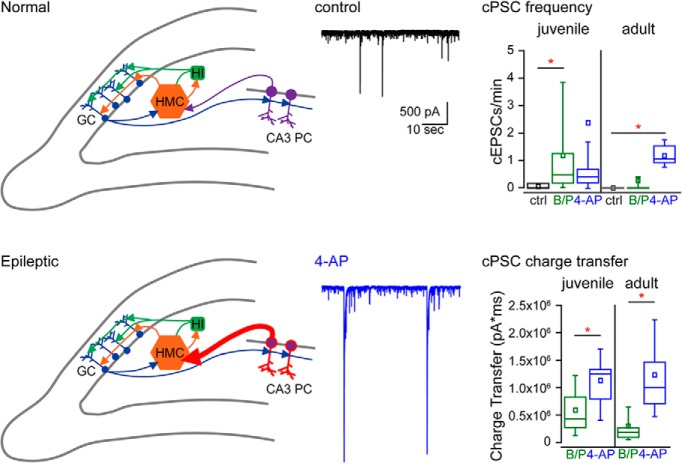

## Significance Statement

Hilar mossy cells (HMCs) constitute a major excitatory principal neuron in the hippocampus with a proposed role in cognitive processing and epilepsy, but many fundamental properties of their excitatory synaptic input have not been characterized. We show that synaptic properties to HMCs from CA3 pyramidal neurons and mossy fibers (MFs) differed in some aspects of their postsynaptic complement of ionotropic glutamate receptors but are surprisingly similar with respect to short-term presynaptic facilitation. In conditions of elevated excitability, the weak but abundant spontaneous input from the CA3 was transformed into large synchronized bursts of excitatory synaptic events. The back-projection from CA3 to mossy cells therefore has the potential to profoundly shape hilar excitability and circuit function in the healthy hippocampal circuit or in pathologic states.

## Introduction

Hilar mossy cells (HMCs) are the only hippocampal glutamatergic neurons that are not a component of the classically defined feed-forward trisynaptic circuit (Andersen et al., 1971; Amaral and Witter, 1989). Nonetheless, these large excitatory neurons scattered in the hilar region are an integral component of memory processes such as pattern separation and completion (Scharfman, 1993a; Buckmaster and Schwartzkroin, 1994; Lisman, 1999; Myers and Scharfman, 2009, 2011). HMC circuit function is transduced through afferent synaptic input from dentate granule neurons (via mossy fibers; MFs), CA3 pyramidal neurons, and to a lesser extent from other HMCs and entorhinal cortex neurons (Ishizuka et al., 1990; Frotscher et al., 1991; Scharfman, 1993a,b, 1994b; Wenzel et al., 1997; Larimer and Strowbridge, 2008).

HMCs have largely escaped the extensive interrogation of synaptic function conducted for analogous glutamatergic synapses in the hippocampus (Scharfman, 2016). MF synapses on HMCs are structurally and functionally similar to those found on CA3 pyramidal neurons, in that the boutons envelope thorny excrescence-like complex spines on HMC dendrites (Lysetskiy et al., 2005; Nahir et al., 2007; Scott et al., 2008) and, in rats, appear to express similar short- and long-term synaptic plasticity (Lysetskiy et al., 2005). Unlike their connections with CA3 pyramidal neurons, however, MFs also form smaller excitatory synapses on distal simple spines of HMCs (Frotscher et al., 1991; Acsády et al., 1998), raising the possibility that heterogeneous synaptic signaling might be transduced by single MFs. Pre- and postsynaptic functional parameters of CA3-HMC synapses have not been studied in detail (Scharfman, 2007, 2016), despite the intermediary role of these connections in retrograde flow of activity from CA3 to the dentate (Scharfman, 1994b; especially in models of epilepsy Scharfman, 1994a; Scharfman et al., 2001). Paired recordings in rats demonstrated that CA3 pyramidal neurons make monosynaptic connections with HMCs, and that CA3-HMC inputs are sparse and relatively weaker than MF-HMC synapses (Scharfman, 1993a,b, 1994b). CA3-HMC synaptic potentials from prior recordings displayed no facilitation and had a high failure rate, which, along with histologic evidence that these synapses are located on the distal HMC dendrite suggest that the CA3 back-projection plays a limited role in driving HMC activity (Ishizuka et al., 1990; Frotscher et al., 1991; Kunkel et al., 1993; Scharfman, 1993a,b; Li et al., 1994; Scharfman, 1994b). In short, we know comparatively little about excitatory synapses in HMCs despite their relevance to cognitive processing (Scharfman, 2016).

Adaptations in HMC synaptic input might contribute to hyperexcitability in epilepsy. Hilar neurons degenerate to varying degrees in models of temporal lobe epilepsy and in the human disease (Margerison and Corsellis, 1966; Sloviter, 1987; Blümcke et al., 2000; Zhang et al., 2015), and consequent adaptations in synaptic connectivity and function could exacerbate dentate excitability (Sloviter et al., 2003; Santhakumar et al., 2005). Synchronized spiking in CA3 propagates to the hilus and thence to the DG in epileptic animals or following application of GABA_A_ receptor antagonists, suggesting that CA3-HMC synapses have the potential to powerfully entrain HMC spiking under hyperexcitable conditions (Scharfman, 1994a).

This study compares synaptic properties of glutamatergic inputs to HMCs from the DG and CA3 in mice. The chief distinctions we found were that CA3-HMC synaptic currents contained a proportionally larger NMDA receptor component to the EPSC and that kainate receptors were selectively localized to MF inputs, as occurs in CA3. Short-term plasticity of MF-HMC synapses was markedly lower than that at CA3 pyramidal cell synapses. Finally, we determined that synchronized CA3 firing is effectively transmitted to HMCs in the form of large amplitude compound postsynaptic currents in HMCs in both *in vitro* and *in vivo* epilepsy models. Our data demonstrates that the balance between excitatory drive to HMCs arising from CA3 and DG changes in the hyperexcitable hippocampus.

## Materials and Methods

### Acute slice preparation

Acute hippocampal slices were prepared from juvenile (P16-P24; average age, P20) or adult (P45-85; average age, P66) C57bl/6 mice of either gender, in accordance with Institutional Animal Care and Use Committee-approved protocols. Mice were transcardially perfused with ice-cold sucrose-rich slicing ACSF containing 85 mM NaCl, 2.5 mM KCl, 1.25 mM NaH_2_PO_4_, 25 mM NaHCO_3_, 75 mM sucrose, 25 mM glucose, 10 µM DL-APV, 100 µM kynurenate, 0.5 mM Na L-ascorbate, 0.5 mM CaCl_2_, and 4 mM MgCl_2_, and oxygenated and equilibrated with 95% O_2_/5% CO_2_. Following perfusion, mice were quickly decapitated and horizontal slices (350 µm) were prepared using a Leica VT1200S vibratome (Leica Biosystems) in sucrose-ACSF. Slices were transferred to a holding chamber containing sucrose-ACSF warmed to 30°C and slowly returned to room temperature over the course of 15-30 min. Slices were then transferred to oxygenated ACSF at room temperature containing 125 mM NaCl, 2.4 mM KCl, 1.2 mM NaH_2_PO_4_, 25 mM NaHCO_3_, 25 mM glucose, 2 mM CaCl_2_, and 1 mM MgCl_2_ and maintained under these incubation conditions until recording.

### Electrophysiological recordings

Slices were transferred to a recording chamber and continuously perfused with oxygenated ACSF at room temperature (25°C). HMCs were visually identified using an upright microscope (Axioskop 2 FS Plus, Zeiss; or BX51WI, Olympus) and subsequently used for whole-cell patch clamp recordings with a MultiClamp 700A or 700B amplifier (Molecular Devices). Borosilicate glass recording electrodes had tip resistances of 4-7 MΩ and were filled with an internal recording solution containing 95 mM CSF, 25 mM CsCl, 10 mM Cs-HEPES, 10 mM Cs-EGTA, 2 mM NaCl, 2 mM Mg-ATP, 10 mM QX-314, 5 mM TEA-Cl, and 5 mM 4-aminopyridine (4-AP), 0.2% (w/v) biocytin, and adjusted to pH 7.3 with CsOH. Whole-cell voltage clamp recordings from HMCs of AMPA receptor-mediated spontaneous EPSCs (sEPSCs), and evoked EPSCs were made at -80 mV. Series resistance was continuously monitored and compensated 60-70%. Access resistance was monitored continuously throughout the duration of evoked experiments and between blocks during sEPSC recordings. Those experiments in which the access resistance changed by >20% were not included in the data analyses. Access resistances ranged between 5 and 25 MΩ, with an average access resistance of 13.2 ± 0.3 MΩ. Protocols were executed using pClamp9 or pClamp10 software (Molecular Devices) and an A310 Accupulser driving a D53 constant current isolated stimulator (Digitimer). Analysis was performed using Clampfit 10.2 (Molecular Devices) or MiniAnalysis (Synaptosoft).

MF-HMC EPSCs were evoked by electrical stimulation in the subgranular zone using monopolar glass electrodes at a mean stimulus intensity of 62 ± 6 μA with a pulse duration of 20 µs and had mean synaptic latencies of 2.5 ± 0.1 ms in juvenile mice (*n* = 15) and 3.1 ± 0.2 ms in adult mice (*n* = 17). A total of 1 µM DCG-IV was applied at the end of the recordings to verify MF identity (with an inclusion criterion of >65% reduction in amplitude). CA3-HMC EPSCs were evoked by electrical stimulation in the CA3 pyramidal cell layer at a mean intensity of 48 ± 4 μA with a pulse duration of 20 µs and had a mean synaptic latency of 3.6 ± 0.2 ms in juvenile mice (*n* = 24) and 3.7 ± 0.3 ms in adult mice (*n* = 17). Both types of recordings were performed in ACSF containing 10 µM bicuculline and 50 µM picrotoxin. External solution in CA3-HMC EPSC recordings included 1 µM DCG-IV to reduce contamination from MF inputs. The paired-pulse (PP) ratio was calculated from peak EPSC amplitudes in response to a pair of electrical stimuli with a 40 ms interstimulus interval. The tau of the EPSC decay was derived from a weighted average of a dual-component exponential fit of the falling phase of the EPSC. NMDA/AMPA ratios were calculated based on the peak AMPAR-mediated EPSC amplitude measured at -80 mV, and the NMDAR-mediated EPSC component measured 40 ms following electrical stimulation at a +40 mV holding potential, respectively. Unitary EPSCs (uEPSCs) were evoked at MF-HMC or CA3-HMC synapses following equimolar substitution of extracellular Ca^+^ with strontium (Sr^+^) in ACSF (Bekkers and Clements, 1999). uEPSCs were manually identified within a time window of 50-350 ms following the electrical stimulus in 15 traces for each recording. Events smaller than 5 pA or with a charge transfer smaller than 200 pA/ms were excluded from the analysis. Spontaneous PSCs were identified and measured in 60 s blocks for each drug condition. Events smaller than 20 pA or with a charge transfer smaller than 200 pA/ms were excluded from the analysis. Compound PSCs (cPSCs) were identified based on a combination of peak amplitude (>1 nA), number of distinct peaks (≥2 within 100 ms), and total charge transfer (>100,000 pA/ms within 2 s from the start of the event).

In a subset of experiments, CA3 was dissected away from the DG to sever the connection between CA3 pyramidal neurons and HMCs. In these experiments, a single cut was made between the two blades of the dentate gyrus using a razor blade. This cut resulted in complete dissection of CA3 from the hilus, however in a minority of slices, the most proximal portion of CA3c was outside of the cut and remained connected to the hilus.

### Intrahippocampal kainic acid (KA) injection

Eight-week-old C57bl/6 mice were anesthetized with 5% inhaled isoflurane and placed in a stereotaxic frame (Stoelting), after which the level of anesthetic was decreased to 1-2% for the remainder of the procedure. Mice were injected unilaterally with either 100 nl sterile saline solution (0.9% saline) or 100 nl KA (20 mM KA diluted in saline solution) into the dorsal hippocampus using a 5 μl Hamilton syringe (injection coordinates relative to bregma: 2.3 mm posterior, 1.3 mm lateral, 1.7 mm deep; Hubbard et al., 2016). The injection syringe was maintained in the hippocampus for 5 min following the injection and then slowly removed. Mice were administered subcutaneous saline solution and consistently monitored following injections. All mice that received KA injections experienced status epilepticus, defined as three or more hours of continuous seizures classified as stage 3-5 on a modified Racine Scale (Racine, 1972).

### EEG recordings and HMC recordings following KA injection

One week following intrahippocampal injection, EEG and EMG electrodes (Pinnacle Technology) that allow two channel EEG and one channel EMG recording were implanted into three KA-injected mice and three saline-injected mice as described previously (Heuermann et al., 2016). Two weeks following intrahippocampal injection and one week following EEG implantation, continual EEG recordings were obtained for 3-6 d, 24 h/d using PAL8200 software (Pinnacle Technology). Seizures were detected manually by scrolling through 60 s epochs of EEG data and identified as high-frequency sharp-wave activity with a duration of at least 10 s and an amplitude of at least twice the baseline EEG amplitude. HMCs were recorded as described above in acute slices from mice unilaterally injected with either KA or saline (six mice injected with KA, five mice injected with saline).

#### Identification of HMCs

HMCs were selected for recording based on the presence of a large, multipolar soma in the hilus. After achieving whole-cell configuration, HMCs were verified by a large whole-cell capacitance (>45 pF), and high frequency of sEPSCs (>5 Hz). In a subset of recordings, HMCs were filled with biocytin through the recording pipette and identified using *post hoc* streptavidin staining (56 cells were recovered and stained for morphologic identification out of a total of 179 recorded cells filled with biocytin; 55 of 56 were confirmed to be mossy cells morphologically). Acute slices were fixed following recording in 4% paraformaldehyde overnight, then stained with streptavidin (Streptavidin Alexa Fluor 594 conjugate; Life Technologies). Images of filled HMCs were obtained using an Olympus FV10 ASW 3.1 confocal microscope (60× objective; NA: 1.35). HMC identity was verified by the presence of thorny excrescences, or “moss,” on the proximal dendrites. A total of 55 of 56 neurons that were recovered (98%) were positively identified as HMCs based on the presence of thorny excrescences (Scharfman and Myers, 2012).

#### Experimental design and statistical analysis

The number of animals used and the number of cells evaluated are noted in the results section for each experiment. Animals of either sex were used during this study and potential sex-specific differences were evaluated. Unless otherwise noted, unpaired *t* tests were used to determine statistical significance. Mean ± SEM is provided throughout the text. All box and whisker plots are represented as mean, median, interquartile range, and 10-90 percentiles.

## Results

### Relative strengths of CA3-HMC and MF-HMC synapses

**Figure 1.**
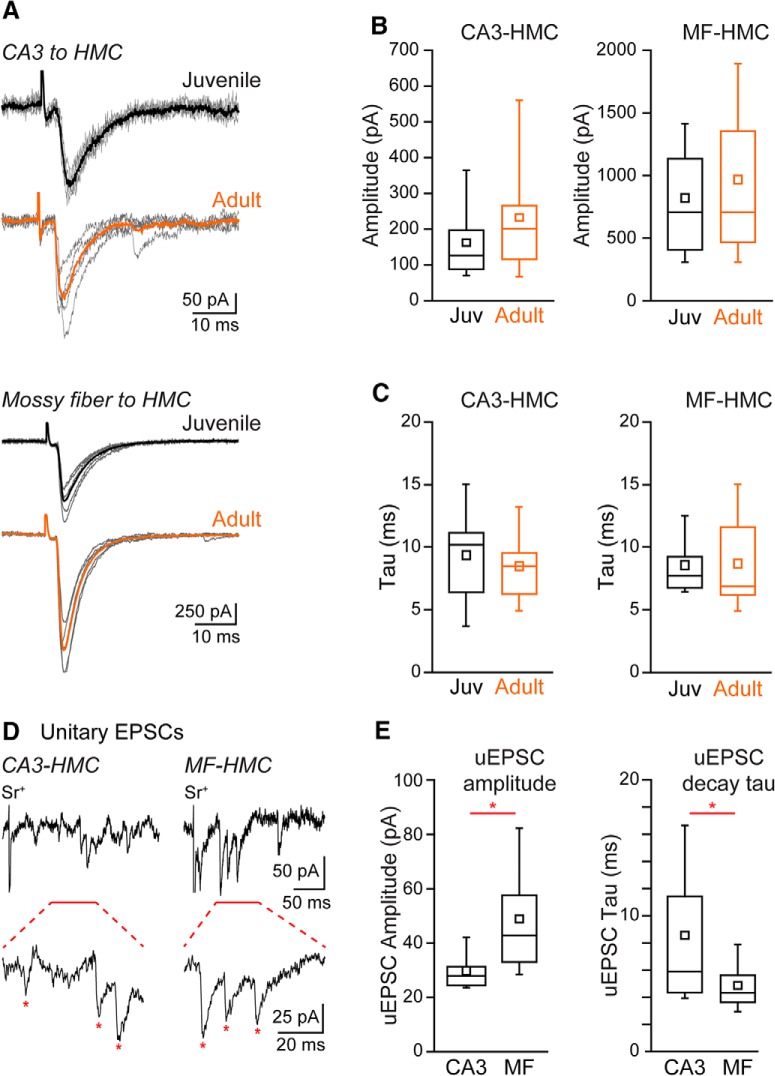
Evoked EPSCs at excitatory HMC afferent synapses. ***A***, top, Example EPSCs at a CA3-HMC synapse from whole-cell recordings of HMCs in acute slices from juvenile and adult animals. Bottom, Example EPSCs at a MF-HMC synapse from whole-cell recordings of HMCs in acute slices from juvenile and adult animals. Gray, individual sweeps; black, average trace juvenile; orange, average trace adult. ***B***, Amplitude of evoked EPSCs in acute slices from juvenile (black) and adult (orange) mice. Left, CA3-HMC synapse. Right, MF-HMC synapse. Decay kinetics in juvenile and adult mice. ***C***, Decay kinetics in juvenile and adult mice. Left, CA3-HMC synapse. Right, MF-HMC synapse. ***D***, Example recordings of evoked uEPSCs from CA3-HMC synapses (left) and MF-HMC synapses (right). ***E***, Amplitude and decay kinetics of uEPSCs at CA3-HMC (left) and MF-HMC synapses (right). *p < 0.05.

The relative strengths of the two primary sources of excitatory synaptic input to HMCs and to what extent those parameters change during maturation is unknown. For those reasons, we compared measures of pre- and postsynaptic function between CA3-HMC and MF-HMC synapses in both juvenile (P16-P24; average age, P20) and adult (P45-P85; average age, P68) mice. Representative traces are shown in Figure 1*A*, with the heavier traces representing the average of the individual events shown in gray. CA3-HMC EPSCs were evoked by electrical stimulation of the CA3c pyramidal layer and had mean amplitudes of 163 ± 20 pA in neurons from juvenile mice (*n* = 27) as compared to 194 ± 36 pA (*n* = 24) in mature animals (Fig. 1*A*,*B*). MF-HMC EPSCs were evoked by electrical stimulation of the subgranular zone and had mean amplitudes of 807 ± 101 pA in juvenile mice (*n* = 24) and 961 ± 120 pA in adult mice (*n* = 28); with slightly increased stimulus intensity, EPSCs of >1 nA were frequently recorded (Fig. 1*A*,*B*). All MF-EPSCs in this dataset met our criterion of >65% reduction in amplitude by DCG-IV (1 μM). Decay kinetics of MF-HMC EPSCs were faster on average than those at CA3-HMC synapses, and in neither case was there a difference between neurons in juvenile and adult mice (CA3-HMC EPSC τ_decay_ in juvenile mice: 10.3 ± 1.3 ms, *n* = 8; adult mice: 8.6 ± 0.9 ms, *n* = 12, *p* = 0.58; MF-HMC EPSC τ_decay_ in juvenile mice: 8.6 ± 0.6 ms, *n* = 15; adult mice: 8.7 ± 0.9 ms, *n* = 18, *p* = 0.88; Fig. 1*A*,*C*). There was no difference in the fast or slow components of the decay at CA3-HMC or MF-HMC synapses (CA3-HMC EPSC τ_decay_ in juvenile mice: τ_fast_ 2.5 ± 0.8 ms, τ_slow_ 17.3 ± 4.5 ms, *n* = 8; adult mice: τ_fast_ 4.5 ± 0.8 ms, τ_slow_ 30.6 ± 7.3 ms, *n* = 12, *p* = 0.10 and *p* = 0.10, respectively; MF-HMC EPSC τ_decay_ in juvenile mice: τ_fast_ 4.3 ± 0.7 ms, τ_slow_ 32.7 ± 8.4 ms, *n* = 15; adult mice: τ_fast_ 4.6 ± 0.7 ms, τ_slow_ 38.3 ± 10.2 ms, *n* = 17, *p* = 0.82 and *p* = 0.67, respectively). Thus, on average CA3-HMC EPSCs were of smaller mean amplitude (at roughly equivalent stimulation strengths, *p* < 0.001 both juvenile and adult) compared to MF-HMC EPSCs, and had slightly slower decay kinetics (*p* = 0.07 juvenile, *p* = 0.02 adult). No clear differences in the properties of juvenile and adult EPSCs were noted at either synapse.

To determine the relative quantal amplitude of the two synapses, we measured uEPSCs in HMCs in the presence of 2 mM Sr^+^ to desynchronize vesicular release from juvenile mice (Fig. 1*D*). uEPSC amplitudes were measured during a time window of 50-350 ms following stimulation. CA3-HMC uEPSCs had a mean amplitude of 29 ± 2 pA (*n* = 10 recordings from three animals) and decayed with a time constant of 8.6 ± 1.7 ms (*n* = 9 recordings from three animals; average number of recorded uEPSCs for each CA3-HMC recording was 69 ± 10; Fig. 1*D*,*E*). MF-HMC uEPSCs were of larger mean amplitude, 49 ± 6 pA (*n* = 11 recordings from three animals; *p* = 0.011) and decayed faster with a τ of 4.8 ± 0.6 ms (*n* = 12 recordings from three animals; *p* = 0.032; average number of recorded uEPSCs for each MF-HMC recording was 75 ± 9) than CA3-HMC uEPSCs (Fig. 1*D*,*E*). The amplitude of MF-HMC uEPSCs was similar to those described previously for MF-CA3 synapses (Lawrence et al., 2004; Marchal and Mulle, 2004). These data suggest that our standard stimulus strength in normal ACSF (Fig. 1) evoked fewer inputs from CA3 (∼5) than MFs from DG (∼16).

### Postsynaptic glutamate receptor content at excitatory HMC synapses

MF synapses on CA3 pyramidal neurons have a number of distinct postsynaptic features that are relatively unusual in the CNS, including the expression of postsynaptic kainate receptors (Scharfman and Schwartzkroin, 1988) and a relatively low NMDA/AMPA ratio (Scott et al., 2008); to what extent MF-HMC synapses share these features is unknown. We therefore compared the contributions of different glutamate receptor subtypes to EPSCs at CA3-HMC and MF-HMC synapses. The AMPA receptor component was measured as the EPSC amplitude at -60 mV; the NMDA receptor component as the current amplitude 40 ms after stimulation while holding the HMC at +40 mV.

**Figure 2.**
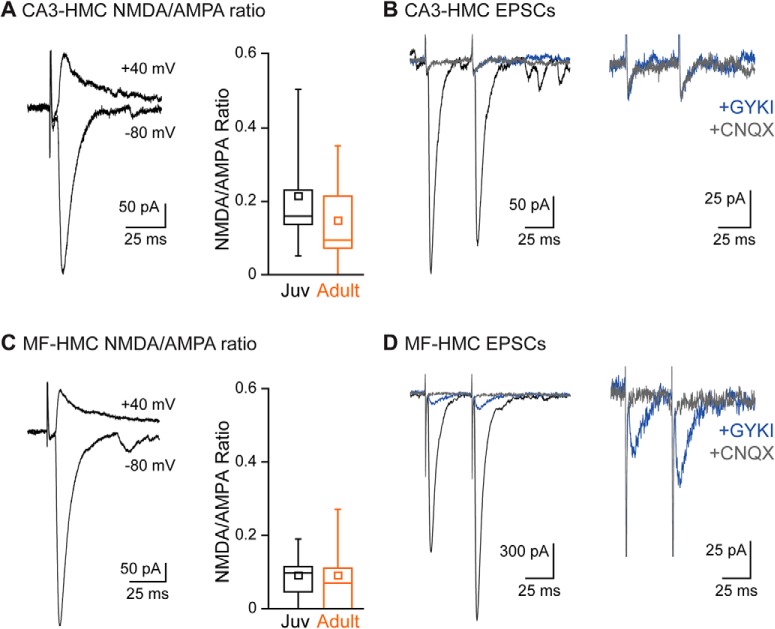
Postsynaptic receptor identity at excitatory HMC afferent synapses. ***A***, Example EPSCs recorded from a CA3-HMC synapse while holding the HMC at -80 mV (lower trace) and +40 mV (upper trace). Population data showing NMDA/AMPA ratios at CA3-HMC synapses in juvenile (black) and adult (orange) mice. ***B***, Example EPSCs recorded at CA3-HMC synapses (black), following addition of GYKI53655 (KAR-mediated component; blue), and following addition of CNQX (glutamate receptors blocked; gray). Expanded traces shown to the right. ***C***, Example EPSCs recorded from a MF-HMC synapse while holding the HMC at -80 mV (lower trace) and +40 mV (upper trace). Population data showing NMDA/AMPA ratios at MF-HMC synapses in juvenile (black) and adult (orange) mice. ***D***, Example EPSCs recorded at MF-HMC synapses (black), following addition of GYKI53655 (KAR-mediated component; blue), and following addition of CNQX (glutamate receptors blocked; gray). Expanded traces shown to the right.

The NMDA/AMPA ratio at CA3-HMC synapses was 0.22 ± 0.06 (*n* = 9 cells from six juvenile animals), with NMDA currents not detected in one out of nine recordings. In adult neurons, the NMDA/AMPA ratio was 0.15 ± 0.04 (*n* = 15 from seven animals), with currents in two recordings lacking a detectable NMDA component (Fig. 2*A*). The AMPA receptor noncompetitive antagonist GYKI 53655 completely eliminated EPSCs at CA3-HMC synapses (*n* = 7), suggesting that postsynaptic kainate receptors are absent at this synapse (Fig. 2*B*). NMDA/AMPA ratios at MF-HMC synapses were lower than those at CA3-HMC synapses and a larger proportion lacked a detectable NMDA EPSC (MF-HMC: juvenile mice, 0.09 ± 0.02, *n* = 14 from 10 animals, with NMDA components not detected in two recordings; adult mice, 0.09 ± 0.03, *n* = 16 from nine animals, no NMDA component in six recordings; Fig. 2*C*). MF-EPSCs clearly contained a kainate receptor EPSC (EPSC_KA_); addition of GYKI 53655 revealed a small residual current with kinetics similar to but somewhat faster than those previously recorded at MF-CA3 synapses (EPSC_KA_ amplitude: 165 ± 34 pA; τ_decay_ was 25.1 ± 6.2 ms, *n* = 29 from 13 animals; Fig. 2*D*; Sloviter, 1989). HMCs therefore parallel CA3 pyramidal neurons in their specific localization of postsynaptic kainate receptors to MF but not at CA3 collateral synapses.

### Short-term plasticity of excitatory HMC synapses

**Figure 3.**
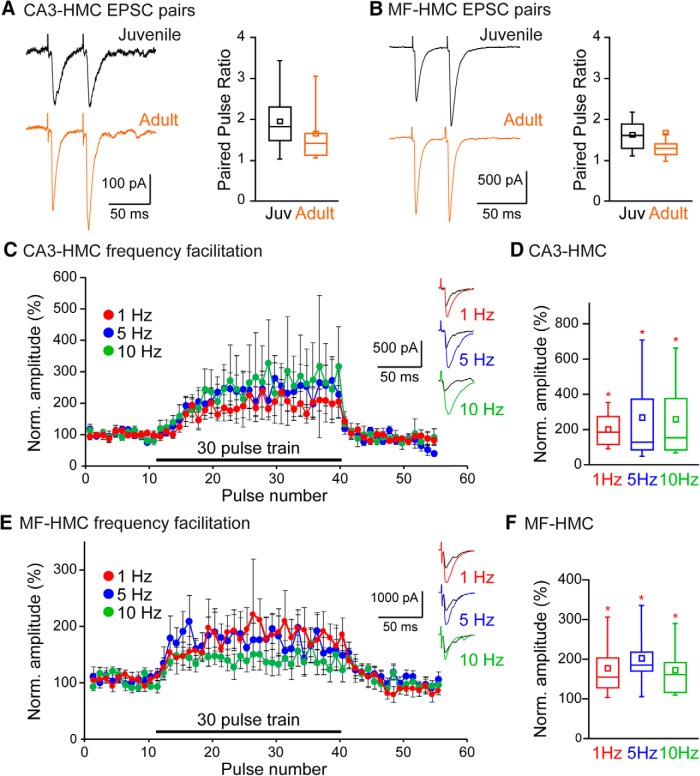
Short-term plasticity at excitatory HMC afferent synapses. ***A***, Example traces showing EPSCs at CA3-HMC synapses in response to paired stimuli 40 ms apart from juvenile (black) and adult (orange) mice. Population data showing paired pulse ratio at CA3-HMC synapses for paired stimuli at a 40 ms interval in juvenile and adult mice. ***B***, Example traces showing EPSCs at MF-HMC synapses in response to paired stimuli 40 ms apart from juvenile (black) and adult (orange) mice. Population data showing paired pulse ratio at MF-HMC synapses for paired stimuli at a 40 ms interval in juvenile and adult mice. ***C***, Population data showing the time course of facilitation at CA3-HMC synapses during a train of 30 stimuli at different stimulation frequencies. Example traces showing baseline EPSC amplitude (black) and EPSC amplitude during stimulus trains at 1 Hz (red), 5 Hz (blue), and 10 Hz (green) frequencies. ***D***, EPSC facilitation at CA3-HMC synapses given trains of stimuli at different frequencies. Average facilitation for each cell measured from pulse number 15-20. ***E***, Population data showing the time course of facilitation at MF-HMC synapses during a train of 30 stimuli at different stimulation frequencies. Example traces showing baseline EPSC amplitude (black) and EPSC amplitude during stimulus trains at 1 Hz (red), 5 Hz (blue), and 10 Hz (green) frequencies. ***F***, EPSC facilitation at MF-HMC synapses given trains of stimuli at different frequencies. Average facilitation for each cell measured from pulse number 5-10. *p < 0.05.

Excitatory synapses on CA3 pyramidal neurons are strikingly divergent in their presynaptic function; MF boutons are unusual in their capability to undergo rapid and profound increases in release probability in response to elevated frequencies of stimulation, which in part underlies their proposed role as “detonator” synapses (Henze et al., 2000), whereas CA3 collateral synapses are more conventional in their synaptic function. To determine whether these differential properties are shared by synapses on HMCs, two forms of short-term plasticity, PP and low-frequency facilitation, were compared in whole-cell HMC recordings. Paired stimuli with a 40 ms interval (PP40) facilitated EPSC amplitudes at both synapses to an equivalent degree [CA3-HMC: juvenile, 2.0 ± 0.1, *n* = 26 from 10 animals; adult, 1.7 ± 0.2, *n* = 22 from 13 animals, *p* = 0.19 (Fig. 3*A*); MF-HMC: juvenile, 1.7 ± 0.1, *n* = 24 from eight animals; adult, 1.7 ± 0.4, *n* = 17 from 13 animals, *p* = 0.90 (Fig. 3*B*)].

To determine how these excitatory synapses responded to longer trains of lower frequency stimuli, we evoked EPSCs at 1, 5, and 10 Hz in trains of 30 stimuli. Again, there was very little difference in presynaptic facilitation between the two synapses (CA3-HMC facilitation: 1 Hz, 203 ± 32%, *n* = 11, *p* = 0.010; 5 Hz: 266 ± 68%, *n* = 15, *p* = 0.014; 10 Hz: 258 ± 70, *n* = 13, *p* = 0.028; paired *t* tests vs pre-tetanus control amplitudes; Fig. 3*C*,*D*). EPSCs at MF-HMC synapses facilitated to a similar degree in response to trains of stimuli (1 Hz: 176 ± 24%, *n* = 11, *p* = 0.010; 5 Hz: 201 ± 27%, *n* = 10, *p* = 0.004; 10 Hz: 172 ± 25%, *n* = 10, *p* = 0.005; Fig. 3*E*,*F*). The relatively modest facilitation at these low frequencies observed at MF-HMC synapses was surprising given the relatively large facilitation seen at anatomically similar MF-CA3 synapses (MF-CA3: 1 Hz, 313 ± 34%, *p* = 0.014, 5 Hz: 525 ± 270%, *p* = 0.015, 10 Hz, 661 ± 426%, *p* = 0.0.018, *n* = 3 from two animals; Salin et al., 1996; Contractor et al., 2001) and a previous report that concluded MF synapses on the two types of cells exhibited comparable degrees of short-term plasticity (Lysetskiy et al., 2005).

### cPSCs in HMCs are evoked by application of proconvulsants in acute slices

**Figure 4.**
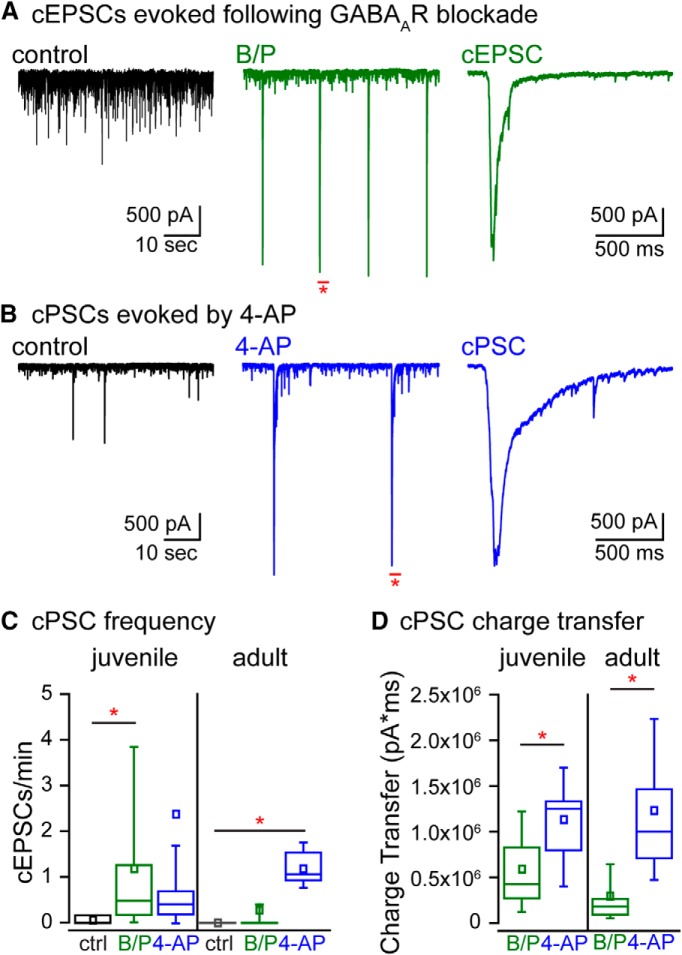
Pharmacological disruption of the hippocampal network leads to cPSCs in HMCs. ***A***, Example traces showing spontaneous synaptic activity in a HMC in ACSF (black), and following addition of bicuculline and picrotoxin (B/P: GABA_A_R antagonists; green). Expanded portion of the trace on the right shows a cEPSC recorded following addition of bicuculline and picrotoxin. ***B***, Example traces showing spontaneous synaptic activity in a HMC in ACSF (black), and following addition of 4-AP (blue). Expanded portion of the trace on the right shows a cEPSC recorded following addition of 4-AP. ***C***, cPSC frequencies in different pharmacological conditions in HMCs recorded in slices from juvenile (left) and adult (right) mice. ***D***, cPSC charge transfers recorded in different pharmacological conditions in slices from juvenile (left) and adult (right) mice.

In our recordings of evoked EPSCs, we noted the presence of large-amplitude compound EPSCs (cEPSCs) that occurred spontaneously. These bore similarities to burst discharges previously recorded from epileptic rats, which were proposed to arise from synchronized firing in the highly recurrent CA3 network propagating through the hilus and to dentate granule cells (Scharfman, 1994a; Myers and Scharfman, 2009, 2011; Scharfman et al., 2001). To determine whether the cEPSCs in our recordings were indeed manifestations of CA3-driven synchronized firing, we recorded sEPSCs in HMCs and compared the frequency of compound bursts in control and disinhibited conditions in neurons from both juvenile and mature animals. cEPSCs were defined as superimposed bursts of sEPSCs with a peak burst amplitude >1 nA, ≥2 superimposed EPSCs within 100 ms, and a total charge transfer of >100,000 pA/ms within 2 s from the start of the event; Fig. 4*A*,*B*). Application of GABA receptor antagonists (bicuculline and picrotoxin) resulted in cEPSCs in juvenile HMCs with a mean charge transfer 596 ± 96 nA/ms (*n* = 180 events from 18 cells from 12 animals), composed of multiple discrete EPSC peaks, lasted for hundreds of milliseconds before returning to baseline and were eliminated by application of CNQX (*n* = 3 from two animals; data not shown) and, thus, likely arose from summed AMPAR-mediated EPSCs (HMCs in 10 of 12 disinhibited slices from seven animals displayed cEPSCs; Fig. 4*A*,*C*,*D*). Compound bursts of synaptic events (cPSCs, comprised of both IPSCs and EPSCs) also were recorded when inhibition was intact but intrinsic neuronal excitability was enhanced with the proconvulsant 4-AP (cPSC frequency in ACSF: 0.07 ± 0.03 cPSC/min; in 4-AP: 2.39 ± 2.02 cPSC/min, *p* = 0.050; HMCs in 9 of 11 slices from five animals displayed cPSCs after addition of 4-AP; in disinhibited slices: 1.20 ± 0.49 cEPSC/min; Fig. 4*B-D*). Surprisingly, application of muscarinic acetylcholine agonist pilocarpine did not lead to the generation of cPSCs in HMCs (no cPSCs in three slices from two animals). cPSCs were somewhat rhythmic in their occurrences, in that the interevent-intervals (IEIs) had a low coefficient of variation (average CV of IEIs in disinhibited slices: 0.45 ± 0.19, *n* = 7; CV of IEIs in 4-AP: 0.49 ± 0.17, *n* = 9). Thus, cPSCs in HMCs were evoked by hippocampal disinhibition or broad depolarization, but not by activation of muscarinic acetylcholine receptors.

To determine whether mice exhibit a maturational difference in generation of compound synaptic currents, their frequency in recordings from slices from adult mice was compared to the previous data from juvenile neurons. cEPSCs in the presence of GABA receptor antagonists were observed only rarely in slices from adult mice (two of 13 recordings from nine adult mice exhibited cESPCs at a frequency of 0.29 ± 0.26 per minute), which was proportionally less common than juvenile HMCs (10 of 12 recordings from seven animals) and less frequent (1.20 ± 0.49 cEPSC/min in those HMCs with events, *p* = 0.047; Fig. 4*C*). Although cEPSCs were rarely observed in recordings from adult neurons, the charge transfer in those that were generated was similar to that in juvenile neurons [280 ± 105 nA/ms in adult mice, *n* = 38 events from 13 cells from nine animals (Fig. 4*D*); as compared to 596 ± 96 nA/ms in juvenile mice, *n* = 180 events from 18 cells from 12 animals]. Thus, the hippocampal circuit in the adult mouse appears less prone to pathologic synchrony in disinhibited conditions than in juvenile mice. Elevation of network excitability with 4-AP, in contrast, led to the generation of cPSCs in acute slices from both juvenile and adult mice with an occurrence and frequency that was not statistically different from recordings in juvenile mice (six of six recordings from three mice with a frequency of 1.18 ± 0.19 cPSCs/min in adult mice; Fig. 4*C*). cPSCs evoked by 4-AP application in acute slices prepared from adult mice had a charge transfer of 1234 ± 376 nA/ms (*n* = 88 events), which was similar to 1177 ± 224 nA/ms in slices from juvenile mice (*n* = 158 events; Fig. 4*D*). The greater charge transfer we saw for cPSCs in 4-AP compared to cEPSCs likely reflects the additional contribution of inhibitory PSCs, which are depolarizing in our low chloride internal solution. In summary, hippocampal slices from juvenile and adult mice can generate cPSCs that are qualitatively similar, but slices from adult mice are less likely to develop cEPSCs in disinhibited conditions than are slices from juvenile mice.

**Figure 5.**
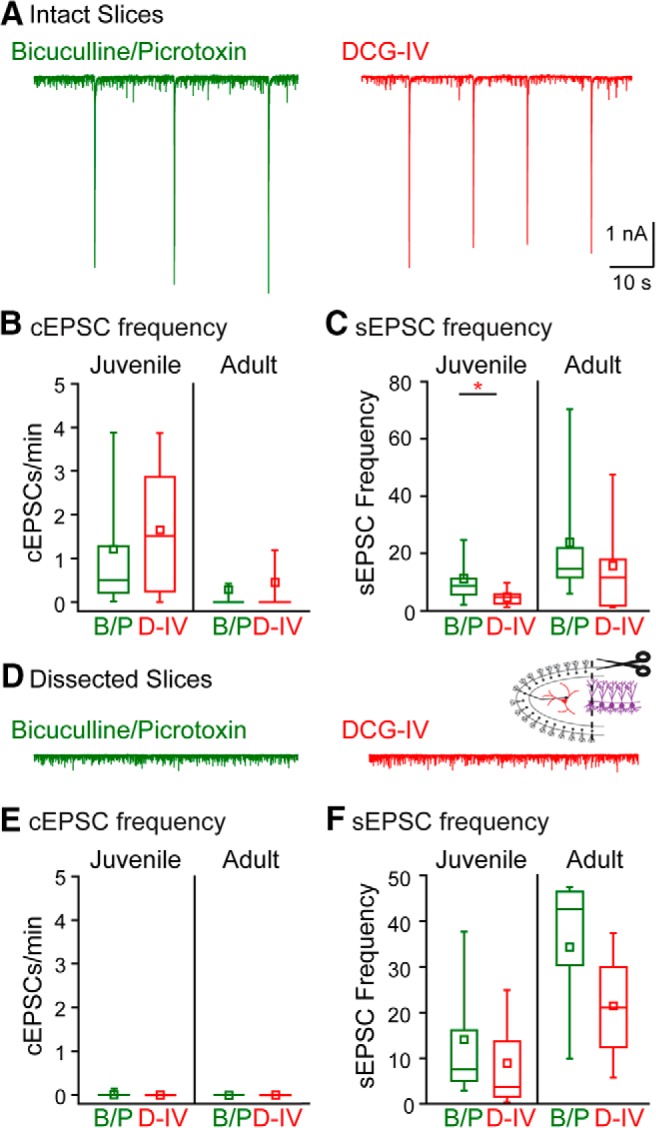
cPSCs propagate to HMCs from CA3. ***A***, Example traces showing cPSCs recorded in HMCs in the presence of bicuculline and picrotoxin (green) and after addition of DCG-IV (red). ***B***, cEPSC frequency in different pharmacological conditions in HMCs from juvenile (left) and adult (right) mice. ***C***, sEPSC frequency in different pharmacological conditions in HMCs from juvenile (left) and adult (right) mice. ***D***, Example traces showing lack of cPSCs in slices where CA3 was dissected away from the hilus. ***E***, cEPSC frequency in HMCs from juvenile (left) and adult (right) mice. ***F***, sEPSC frequency in HMCs from juvenile (left) and adult (right) mice.

We differentiated the DG or CA3 as the origin of synchronized input that generated compound bursts by either reducing release probability at MF synapses with DCG-IV or by lesioning slices at the CA3-hilar border. In slices disinhibited with GABA_A_ receptor antagonists, bath application of DCG-IV did not alter cEPSC frequency in HMC recordings from juvenile or adult mice (juvenile: 1.65 ± 0.35 cEPSCs/min, *n* = 18 from 12 animals, *p* = 0.40 compared to data from Fig. 4*C*; adult: 0.43 ± 0.28 cEPSCs/min, *n* = 17 from 11 animals, *p* = 0.73; Fig. 5*A*,*B*). To test whether cEPSCs originated in CA3, acute slices were prepared, in which a cut was made between the tips of the two blades of the dentate gyrus to separate the hilus from CA3 pyramidal neurons. No cEPSCs were observed in HMC recordings from cut slices (*n* = 8 recordings from four animals; Fig. 5*D*,*E*). In contrast to cEPSCs, individual synaptic currents (sEPSCs) were reduced by application of DCG-IV in recordings from slices prepared from both juvenile and adult mice (juvenile: control, 11.0 ± 2.2 Hz; DCG-IV, 4.85 ± 0.85 Hz; *n* = 13 from seven animals, *p* = 0.004; adult: control, 23.8 ± 8.5 Hz; DCG-IV, 13.6 ± 5.1 Hz; *n* = 11 from seven animals, *p* = 0.03). Separation from CA3 did not measurably alter HMC sEPSC frequencies compared to recordings from uncut slices (juvenile: in bicuculline/picrotoxin, 14.1 ± 5.7 Hz; DCG-IV, 9.0 ± 3.9 Hz; *n* = 8 from four animals, *p* = 0.47; adult: in bicuculline/picrotoxin, 34.6 ± 10.2 Hz; DCG-IV, 21.1 ± 10.3 Hz; *n* = 4 from two animals, *p* = 0.41; Fig. 5*C*,*F*). These data therefore establish the CA3 as the origin of the synaptic bursts and are consistent with the proposal from earlier studies that synchronized firing in the CA3 is effectively propagated to mossy cells in the hilus (Scharfman, 1994a; Scharfman et al., 2001).

### cPSCs propagate to HMCs in epileptic mice

**Figure 6.**
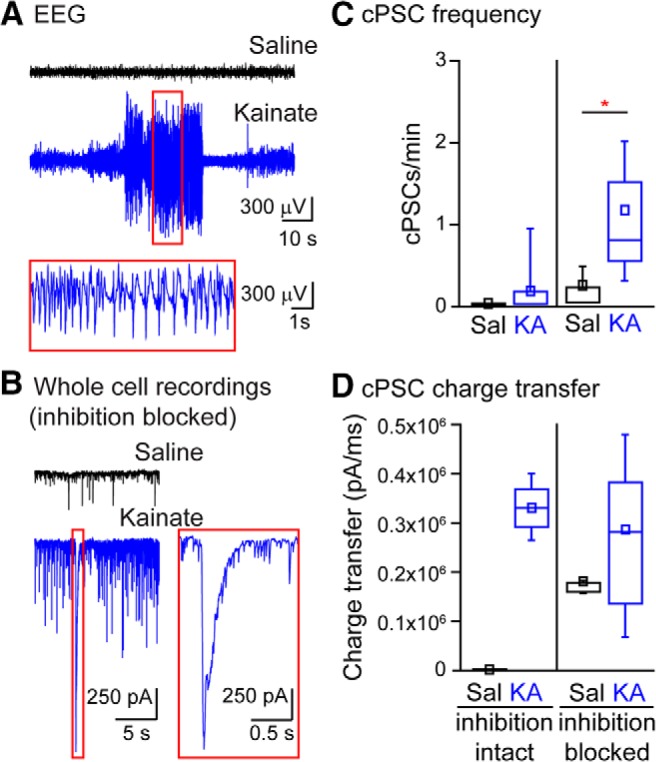
cPSCs are present in mice with chronic, spontaneous seizures. ***A***, Example EEG traces from mice injected intrahippocampally with saline (black) or KA (blue). Example from KA-injected mouse shows a spontaneous seizure with inset showing 10 s of the seizure event. ***B***, Whole-cell recordings of HMCs in acute slices from mice injected intrahippocampally with saline (black) or KA (blue) in the presence of GABA_A_R antagonists bicuculline and picrotoxin. Expanded portion of the trace from the KA-injected mouse shows a cEPSC (right). ***C***, cPSC frequencies in HMCs in acute slices from intrahippocampally injected mice shows an increase in cPSCs in KA-injected mice following addition of bicuculline and picrotoxin. ***D***, cPSC charge transfers in HMCs from intrahippocampally injected mice. Note: no cPSCs were observed in saline-injected mice before the addition of bicuculline and picrotoxin.

To determine whether HMC cPSCs are present in mice with spontaneous convulsions, we induced chronic, spontaneous seizures in adult mice by intrahippocampal KA injection. Adult mice were injected with 100 nl of either saline or 20 mM KA. Two weeks following injections, all six KA-injected mice displayed visible seizures; none of the five control mice had seizures. EEG recordings from three mice in each condition confirmed the presence of electrographic seizures in KA- but not saline-injected mice; representative traces are shown in Figure 6*A*. We then prepared hippocampal slices from the mice and recorded cPSCs and cEPSCs from HMCs. Compound bursts of synaptic events were present before the addition of GABA_A_ receptor antagonists in three of 12 HMCs from KA-injected mice (at a frequency of 0.05 ± 0.02 events/min, cPSC charge transfer: 165 ± 18 nA/ms); none of the recordings from saline-injected mice contained cPSCs (Fig. 6*B-D*). Disinhibition of the slices elicited cEPSCs in 11 of 12 HMCs from mice injected with KA but only three of 12 saline-injected mice; in those neurons with cEPSCs, the frequency of events was higher in epileptic group (KA-injected frequency: 1.1 ± 0.3 events/min; saline-injected frequency: 0.20 ± 0.14 events/min, *p* = 0.01; Fig. 6 *B*,*C*). There was no difference in sEPSC frequency or amplitude in HMC recordings from KA- or saline-injected mice (KA group: 13.8 ± 2.4 Hz, amplitude: 71 ± 7 pA, *n* = 12; saline group: 11.5 ± 2.4 Hz, 66 ± 9 pA, *n* = 11). Thus, intrahippocampal KA injection leads to increased bursts of synchronized input to HMCs arising from hyperexcitability of the CA3 network.

## Discussion

The current study was motivated by the surprising paucity of information regarding fundamental components of excitatory synapses on HMCs under physiologic and pathologic conditions. In particular, back-projections from CA3 to HMCs were described several decades ago but have not been examined functionally to the same extent as other synaptic contacts made by CA3 pyramidal neuron collaterals (Scharfman, 2016). This report provides new information on the receptor complement and short-term plasticity at CA3-HMC synapses and contrasts those parameters with MF inputs to HMCs. We also determined that synchronized firing of CA3 neurons produces strongly depolarizing bursts of excitatory events in hyperexcitable conditions, underscoring the potential strength of the back-projection from CA3 to HMCs. Our data provide essential insight into basic aspects of connectivity that will be of use in explorations of the role of this circuit in cognition and in epileptic states.

### The CA3 back-projection to HMCs

The back-projection from CA3 pyramidal neurons to HMCs has been hypothesized to be important for information processing in the hippocampus, providing a means for CA3 neurons to integrate input from perforant path projections and recurrent CA3 networks and to shape dentate excitability (Scharfman, 2007). The CA3 back-projections to the hilus play a crucial role in functions such as pattern completion and pattern separation (Lisman, 1999; Myers and Scharfman, 2009, 2011; Senzai and Buzsáki, 2017). Despite the potential importance of this projection, this synapse is relatively understudied compared to those between other principal neurons in the hippocampus (Scharfman, 1993b, 1994a). We found that CA3 synapses onto HMCs share properties with their analogous synapses on CA3 pyramidal neurons, exhibiting a substantial NMDA receptor component, an absence of postsynaptic kainate receptors, and modest degrees of short-term facilitation at low stimulation frequencies (Debanne et al., 1996; Castillo et al., 1997; Debanne et al., 1998; Perez-Rosello et al., 2011). CA3 pyramidal neurons often fire in bursts of action potentials *in vivo* (Tropp Sneider et al., 2006); facilitation at CA3-HMC synapses therefore suggests that CA3 pyramidal neurons have the potential to shape HMC activity during burst firing despite their modest unitary conductances. NMDA receptor-mediated currents at this synapse were prominent compared to MF synapses, and thus the back-projection from CA3 to HMCs is primed for conventional NMDA receptor-dependent long-term potentiation. The precise mechanisms of activity-dependent potentiation, such as whether they exhibit the unusual form of spike-timing-dependent plasticity discovered recently at CA3-CA3 synapses (Mishra et al., 2016), remain to be explored. *In vivo* work suggests that HMCs are highly active during behavior (Henze and Buzsáki, 2007; Danielson et al., 2017; Senzai and Buzsáki, 2017), whereas granule cells (Mistry et al., 2011; Danielson et al., 2016) have very sparse firing, suggesting that the back-projection from CA3, rather than the MF input, may be a principal driver of HMC activation *in vivo*.

### Shared and contrasting features of MF-HMC and MF-CA3 synapses

Like MF-CA3 synapses, MF-HMC synapses are formed by MF giant synaptic boutons that are opposed to “thorny excrescences” on proximal dendrites (Frotscher et al., 1991; Kunkel et al., 1993). Recordings at MF-HMC synapses in rat suggest that they are physiologically similar to MF-CA3 synapses (Acsády et al., 1998; Lysetskiy et al., 2005). Surprisingly, in mice, we found that MF-HMC EPSCs did not facilitate to the same degree as those at MF-CA3 synapses (Salin et al., 1996; Henze et al., 2000; Fernandes et al., 2015). In CA3 pyramidal neurons, the MF input has been described as a “conditional detonator”; strong depolarization of pyramidal neurons occurs at higher frequencies or bursts of stimulation, in which the facilitation of MF glutamate release outweighs the feed-forward inhibition mediated by CA3 interneurons (Urban et al., 2001; Henze et al., 2002; Mori et al., 2004). The lower degree of facilitation at HMC synapses suggests that MFs might not serve an equivalent function, although we note that a more robust facilitation in rat recordings was reported previously (Lysetskiy et al., 2005), suggesting there could be species-dependent variability. Anatomic differences between MF-HMC and MF-CA3 synapses also might contribute to these differences: while MF-CA3 synapses are thought to be exclusively at CA3 thorny excrescences, MF-HMC synapses are likely a mixture of similar complex synapses that innervate HMC thorny excrescences but also small terminals that innervate the distal dendrites (Acsády et al., 1998). It is possible that bouton and non-bouton MF synapses express different degrees of short-term plasticity. Alternatively, the relatively low level of facilitation could arise from the absence at MF-HMC synapses of presynaptic kainate receptors (Scott et al., 2008), which increase release probability when activated by endogenous release of glutamate at MF-CA3 pyramidal synapses (Contractor et al., 2001; Schmitz et al., 2001).

With respect to the postsynaptic complement of iGluRs, MF synapses on HMCs were similar to those on CA3 pyramidal neurons. The NMDA/AMPA ratio was low, and the EPSCs contained a kainate receptor component with slow kinetics of activation and decay similar to the canonical MF EPSC_KA_ (Castillo et al., 1997; Mulle et al., 1998). The slow time course of decay of postsynaptic kainate receptors prolongs depolarization with sustained stimulation and alters temporal integration and spike firing in CA3 pyramidal neurons (Sachidhanandam et al., 2009; Pinheiro et al., 2013), and likely subserve the same function at MF synapses in HMCs. The presence of postsynaptic NMDA receptors similarly raises the possibility that MF-HMC synapses might express forms of LTP and metaplasticity that have been discovered only recently at MF-CA3 synapses (Kwon and Castillo, 2008; Rebola et al., 2008; Rebola et al., 2011), in addition to the classic presynaptic form of MF LTP (Zalutsky and Nicoll, 1990; Lysetskiy et al., 2005).

### Synaptic inputs in hyperexcitable conditions

An objective of this study was to determine how synaptic input to HMCs is altered in conditions of hyperexcitability. We found that large-amplitude cEPSCs occurred spontaneously during our recordings, similar to burst discharges recorded in previous work from epileptic rats (Scharfman, 1994a; Scharfman et al., 2001; Myers and Scharfman, 2009, 2011). Synchronized bursting in CA3 occurs in hyperexcitable states (Scharfman, 1994a; McCloskey and Scharfman, 2011), and the development of these bursts is associated with oscillatory rhythms present in both humans with epilepsy and in rodent models (Zijlmans et al., 2009; Jefferys et al., 2012). We confirmed that cEPSCs observed in HMCs originate in CA3 and that these synchronous bursts were enhanced in hyperexcitable conditions, with the generation of compound bursts following disinhibition as well as when the K^+^ channel blocker 4-AP was applied. The back-projection between CA3 and HMCs thus has the potential to powerfully shape hilar excitability through transmission of synchronous activity to the dentate gyrus.

Synchronized burst discharges from CA3 directly impact HMC excitability but also have to potential to converge on interneuron populations that provide inhibitory tone on HMCs. Recent work suggests that CA3 has relatively sparse recurrent connectivity in microcircuits (Guzman et al., 2016). The net effect of increased excitability will therefore likely depend in part on the connectivity and innervation patterns of these small circuits of synchronized CA3 pyramidal neurons, and the direct effect on the DG will be highly dependent on whether a particular microcircuit has its predominant actions on hilar interneuron or HMCs. During hyperexcitable states such as epilepsy where hilar interneuron populations are altered, this network activity is likely altered, changing the balance of excitatory drive to the dentate gyrus. Structural adaptations in one or more populations of hilar neurons also could contribute to hyperexcitability, similar to what occurs in CA1 pyramidal neurons and DGCs in a rodent model of febrile status epilepticus (Patterson et al., 2017).

Earlier work has found that synchronous events in the hippocampus are more prolonged in juvenile mice than in adult mice (Shao and Dudek, 2009). We similarly found differences in cPSCs recorded in HMCs with age as they were readily evoked by network disinhibition in juvenile but not in adult animals. Application of 4-AP, however, led to the generation of cEPSCs in both juvenile and adult mice. There is evidence that 4-AP causes synchronous firing of hippocampal interneurons, particularly hilar interneurons, in acute slices (Fueta and Avoli, 1992; Grosser et al., 2014; Avoli and Jefferys, 2016), suggesting perhaps that these synchronous events are driven in part by altered activity of hilar interneurons and may be a factor in the enhanced seizure susceptibility of immature brains (Rakhade and Jensen, 2009). Since pathologic network synchrony, in the form of cPSCs, was enhanced in adult mice following intrahippocampal KA injection and after the generation of spontaneous seizures, there could be a developmentally enhanced role of hippocampal inhibition in the adult mouse hippocampus that is disrupted following seizures.

No direct evaluation of the CA3-HMC synapse has been performed in the context of epilepsy; however, our results suggest that this synapse may be instrumental in propagating pathologic network activity through the hippocampus during epileptogenesis and be a driving force in HMC degeneration and dysfunction in TLE. HMCs that survive initial seizures might serve as a powerful epileptic focus by relaying pathologic synchronous activity from CA3 through the hilus to the dentate gyrus. We posit that the projection from CA3 to HMCs could play an important role not only in the healthy hippocampal circuit but in pathologic states such as epilepsy.

## Synthesis

Reviewing Editor: Tallie Z. Baram, University of California-Irvine Decisions are customarily a result of the Reviewing Editor and the peer reviewers coming together and discussing their recommendations until a consensus is reached. When revisions are invited, a fact-based synthesis statement explaining their decision and outlining what is needed to prepare a revision will be listed below.

This is a strong and technically accomplished series of experiments. The authors responded very well to previous critiques.

The findings about juvenile vs adult brain are intriguing. The authors might wish to include recent discoveries on age-specific changes in the epileptic DG circuitry (exuberant apical dendritic trees in granule cells) as a potential relevant factor in HMC innervation (Patterson et al.,J Neurosci 2017).
